# QTL Mapping for Gummy Stem Blight Resistance in Watermelon (*Citrullus* spp.)

**DOI:** 10.3390/plants10030500

**Published:** 2021-03-08

**Authors:** Eun Su Lee, Do-Sun Kim, Sang Gyu Kim, Yun-Chan Huh, Chang-Gi Back, Ye-Rin Lee, Muhammad Irfan Siddique, Koeun Han, Hye-Eun Lee, Jundae Lee

**Affiliations:** 1 Vegetable Research Division, National Institute of Horticultural and Herbal Science, Rural Development Administration, Wanju 55365, Korea; lus4434@korea.kr (E.S.L.); greenever@korea.kr (D.-S.K.); kimsg9@korea.kr (S.G.K.); lyr1219@korea.kr (Y.-R.L.); arafay68@yahoo.com (M.I.S.); hke1221@korea.kr (K.H.); 2 Herbal Crop Research Division, National Institute of Horticultural and Herbal Science, Rural Development Administration, Eumseong 27709, Korea; wmelon@korea.kr; 3 Horticultural and Herbal Crop Environment Division, National Institute of Horticultural and Herbal Science, Rural Development Administration, Wanju 55365, Korea; plantdoctor7@korea.kr; 4 Department of Horticulture, Institute of Agricultural Science & Technology, Jeonbuk National University, Jeonju 54896, Korea

**Keywords:** gummy stem blight, high-resolution melting, single nucleotide polymorphism, linkage map, quantitative trait loci

## Abstract

Watermelon (*Citrullus* *lanatus*) is an economically important fruit crop worldwide. Gummy stem blight (GSB) is one of the most damaging diseases encountered during watermelon cultivation. In the present study, we identified quantitative trait loci (QTLs) associated with GSB resistance in an F_2_ population derived from a cross between maternal-susceptible line ‘920533’ (*C. lanatus*) and the paternal-resistant line ‘PI 189225’ (*C. amarus*). The resistance of 178 F_2_ plants was assessed by two different evaluation methods, including leaf lesion (LL) and stem blight (SB). To analyze the QTLs associated with GSB resistance, a linkage map was constructed covering a total genetic distance of 1070.2 cM. QTL analysis detected three QTLs associated with GSB resistance on chromosome 8 and 6. Among them, two QTLs, *qLL8.1* and *qSB8.1* on chromosome 8 identified as major QTLs, explaining 10.5 and 10.0% of the phenotypic variations localizing at same area and sharing the same top markers for both LL and SB traits, respectively. A minor QTL, *qSB6.1*, explains 9.7% of phenotypic variations detected on chromosome 6 only for the SB trait. High-throughput markers were developed and validated for the selection of resistant QTLs using watermelon accessions, and commercial cultivars. Four potential candidate genes were predicted associated with GSB resistance based on the physical location of flanking markers on chromosome 8. These findings will be helpful for the development of watermelon cultivars resistant to GSB.

## 1. Introduction

Watermelon (*Citrullus lanatus*; 2n = 2x = 22) is one of the most important fruit crops, accounting for 11.4% of cultivation area among fruit around the world [1]. Watermelon consists of more than 91% of water and contains functional compounds such as lycopene, citrulline, and β-carotene that contribute in the uptake of a balanced diet and healthy nutrients [2,3].

Gummy stem blight (GSB), caused by *Stagonosporopsis* species pathogen *Didymella bryoniae* (Auersw.) Rehm, causes severe damages to Cucurbit crops, including watermelon. GSB occurs mainly under high temperature and humidity [4,5]. GSB results in small brown spots on the leaves, yellow coloration of the leaves with round or irregular lesions, and greyish-brown stems followed by withering [6]. In particular, leaves and stems tend to dry out and fall early [7], resulting in the rapid advancement of GSB after fruit setting, which leads to watermelon blight before the harvest [8]. The GSB can cause crown blight, severe defoliation, rotting of fruits, and deterioration of the fruits during the transportation and storage, resulting in heavy economic losses. The yield loss could be as high as 100% during the warm and humid season under high disease pressure [9].

In order to control GSB in watermelon, agricultural fungicides should be sprayed prior to the appearance of disease, and crop rotation ought to be performed in case of severe occurrence at the greenhouse or field [9,10]. However, excessive use of chemicals to control GSB can be hazardous to the ecosystem and agricultural safety. Cultural and agronomic controls are expensive and labor-intensive [10]. The development of disease-resistant cultivars can be the best alternative for controlling GSB [11,12]. The watermelon accessions ‘PI 189225’ and ‘PI 271778’ (*C. amarus*), from the US Germplasm Collection of USDA-ARS (United States Department of Agriculture-Agricultural Research Service) have been reported to exhibit GSB resistance [13,14]. Other accessions including, ‘PI 279461’, ‘PI 526233’, and ‘PI 482283’ have also been reported as new resources for GSB resistance [15]. According to Norton et al. [16], the GSB resistance in ‘PI 189225’ was controlled by a single gene, *db*; however, recent research by Gusmini et al. [17] suggested the involvement of multiple genes with environmental factors for GSB resistance. Further studies are required to determine the mode of inheritance associated with resistance to GSB.

Recently, QTL mapping analysis was carried out for resistance to bacterial fruit blotch (BFB), anthracnose race 1, fusarium wilt race 1, and *papaya ringspot virus*-watermelon strain (PRSV-W) using bi-parental mapping populations in watermelon. [18,19,20,21,22]. The quantitative trait loci (QTL) mapping for *Fusarium oxysporum* f. sp. *niveum* race 2 of watermelon has also been carried out [23]. The results of QTL mapping associated with GSB resistance have been reported in various cucurbits. In cucumber, major QTLs related to GSB resistance on leaves and stems were identified on chromosome 5 and 6, respectively [24,25]. Meanwhile, in melon, QTLs conferring resistance of GSB were detected on chromosome 9 [26,27]. The QTL identification linked to GSB (isolate: JS002; JAAS, Nanjing, China) resistance in watermelon were performed using bulked segregant analysis (BSA) [28]. This study detected a QTL (*Qgsb8.1*) on chromosome 8 with an LOD score ranged from 13.6 to 16.4, explaining 31.54 to 32.42% phenotypic variations in two different seasons [28]. Recently, GSB-resistant QTLs were mapped on chromosome 3, 5, and 7 explaining between 6.4 to 21.1% of phenotypic variation [29].

Molecular markers have been developed and deployed for marker-assisted selection in watermelon disease-resistant breeding. Cleaved amplified polymorphic sequence (CAPS) markers and high-resolution melting (HRM) based markers have been reported using ‘Arka Manik’ and ‘PI 254744’ resistant to powdery mildew race 1W in watermelon [30,31]. In addition, CAPS molecular markers related to *zucchini yellow mosaic* virus (ZYMV) resistance were developed in watermelon [32]. The HRM and KASP assays are relatively simple, rapid, and cost-effective approaches compared to other methods such as CAPS, sequence characterized amplified region (SCAR), and simple sequence repeat (SSR) analysis [33]. Recently, KASP assay were developed for GSB resistance in watermelon [28,29]. However, more high-throughput molecular markers are required for efficient prediction of GSB resistance to use in marker-assisted selection programs.

In the present study, QTL analysis was conducted to map resistant loci associated with GSB resistance in watermelon, using next-generation re-sequencing based SNPs. High-throughput molecular markers linked to GSB resistance were developed and validated using watermelon accessions and commercial cultivars. In addition, four potential candidate genes were predicted in QTL regions associated with GSB resistance in watermelon.

## 2. Results

### 2.1. Resistance to GSB in Parents and an F_2_ Population

**Figure 1:**
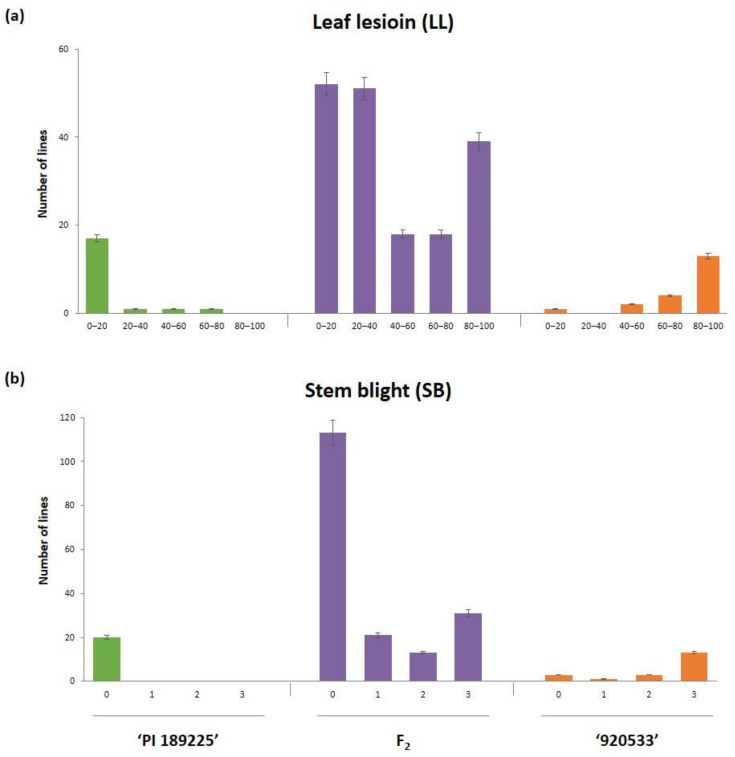
Frequency distribution of disease severity. (**a**) Leaf lesion (LL) and (**b**) stem blight (SB) among parental lines and F_2_ population. Green, orange and purple bar indicates resistant parent ‘PI 189225’, susceptible parent ‘920533’, and an F_2_ population obtained from a cross between ‘920533’ × ‘PI 189225’, respectively. Error bars indicates the standard error (SE).

The average of leaf lesion (LL) of ‘PI 189225’ and ‘920533’ to GSB for the 20 plants was 14.04% and 73.73%, respectively (Figure S1). Both the resistant and susceptible parental lines showed significant difference in disease severity (Student’s *t*-test; *p* < 0.001). According to the combined disease index (DI) scoring, a total of 178 plants in F_2_ population segregated to 65 susceptible and 113 resistant plants, which do not fit into 3:1 (R:S) segregation ratio from Chi-square value of 12.59 at *p* < 0.001 (Table S1). The frequency distribution of LL of the F_2_ population displayed a pattern with two peaks (Figure 1a). Plants of the left peak (resistant) were greater in number than those of the right peak (susceptible). Frequency distribution plot of SB of F_2_ population was drawn to a right-skewed pattern (Figure 1b). The frequency distribution curves and segregation data supported the presence of a quantitative mode of inheritance of resistance to GSB in F_2_ population. These results imply that multiple genes in this population control disease resistance.

### 2.2. SNP Genotyping Using Fluidigm^®^ SNP Type^TM^ Assays

The genotypes of 178 individuals in the F_2_ population were analyzed using previously developed 438 Fluidigm^®^ SNP Type™ assays [34]. Polymorphic SNPs were clustered into three groups in a graph according to the ratios of FEM to HEX fluorescence for individuals of F_2_ population and two parental lines (Figure S2). Red and green-clustered dots indicate homozygous genotypes, and blue-clustered dots indicate heterozygous genotypes (Figure S2). Among these 438 Fluidigm^®^ SNP Type™ assays, 113 polymorphic SNP between the parental lines were selected using Fluidigm^®^ genotyping platform and used for the linkage analysis in F_2_ population (Table 1).

### 2.3. Identification of SNPs Using NGS

In order to develop SNP markers that cover the whole genome, a homozygous susceptible line (‘920533’) and a resistant line (‘PI 189225’) were sequenced with the next generation sequencing method using a Hiseq™ 4000 sequencer (Illumina Inc., San Diego, CA, USA). The raw reads for the parental lines were generated about 17.8 Gbp and 16.8 Gbp, respectively. By trimming the raw data, the sequences were shortened to 13.6 Gbp for ‘920533’ and 13.1 Gbp for ‘PI 189225’. The 320 Mbp (90.13%) and 305 Mbp (85.92%) trimmed data of the resistant and susceptible parental lines was mapped to the reference genome, respectively. The mapped sequences were used to detect SNPs between ‘920533’ and ‘PI 189225’. A total of 6 million raw SNPs were obtained between the parental lines (Table S2) [35].

### 2.4. Development of HRM Markers Using Identified SNPs

Among the total SNPs obtained, 5 million SNPs were selected as homozygous SNPs (Table S2) [35]. Subsequently, 4.3 million SNPs were filtered for A/G, A/C, T/G, and T/C combinations, excluding A/T and G/C. Furthermore, around 2 million SNPs whose flanking sequence (single copy) was found only once in the whole genome were chosen using the basic local alignment search tool nucleotide (BLASTN). Finally, 354,860 SNPs located on the genic region were further selected, and then 888 HRM markers that were evenly distributed on the watermelon genome were developed (Table S2) [35]. Polymorphisms of these HRM markers were tested using two parental lines, the F_1_ plants and 13 randomly selected plants of the F_2_ population. As a result, 84 HRM markers were identified showing polymorphic melting curves between the parents. A total of 88 HRM primer sets, including the 84 newly designed HRM markers and four previously developed HRM markers were used to analyze the genotypes of 178 F_2_ individuals (Table S3 and Figure S3) [36]. Newly designed HRM markers were abbreviated on watermelon gummy stem blight resistance SNPs (WGRS).

### 2.5. Construction of a Watermelon Linkage Map

**Figure 2:**
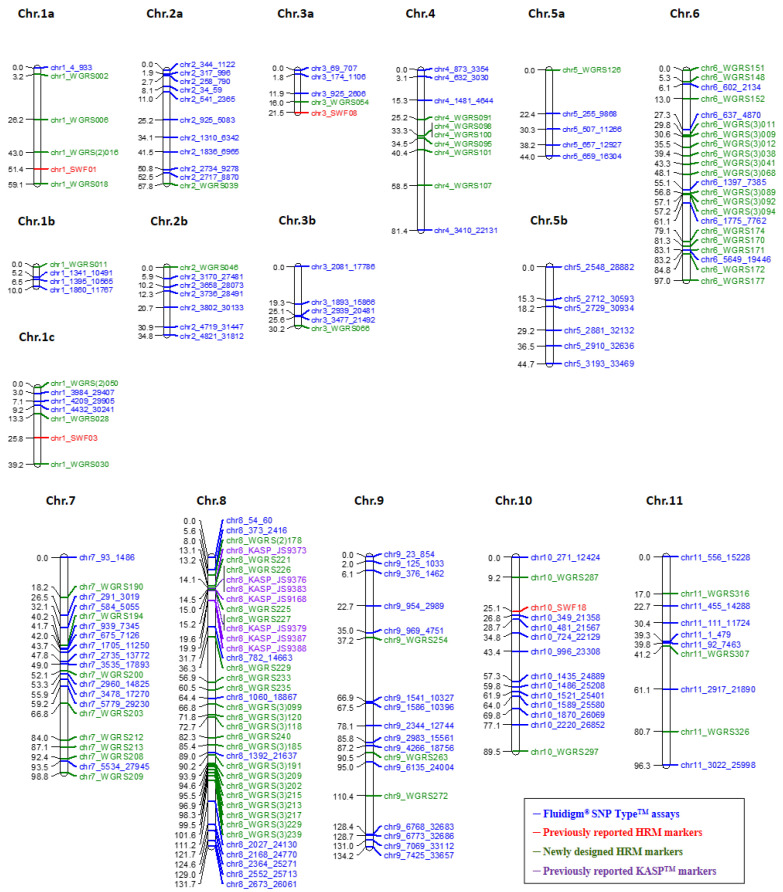
Genetic map of watermelon constructed by SNP markers. Numbers on the left side correspond to the genetic distance in cM from the top of each chromosome. Names on the right side indicate marker name that represents marker type according to color (blue, Fluidigm^®^ SNP Type™ assays; red, previously reported HRM markers; green, newly designed HRM markers; purple, previously reported KASP™ markers).

A genetic linkage map was constructed using 178 F_2_ plants segregating for GSB resistance, and 211 SNP markers. The genetic map was generated using 211 SNP markers consisting of 88 high-resolution melting (HRM), 113 Fluidigm^®^, and 10 recently reported kompetitive allele specific PCR (KASP™) markers [28]. Among them, 188 SNP markers that included 104 Fluidigm^®^ markers, 77 HRM markers, and 7 previously reported KASP™ markers were mapped on 16 linkage groups (LGs) whereas 23 markers remained unmapped (Figure 2 and Figure S4). LGs were named according to chromosomes, as there was a high identity between the genetic map and physical map. The total length of the genetic linkage map was 1070.2 cM where the average length of the linkage groups was 66.89 cM, and the average interval between the markers was 5.69 cM with a mean of 11.8 markers per linkage group (Table 1 and Table S4). The lengths of linkage groups ranged from 10.0 cM to 134.2 cM (Table 1). The number of markers per linkage group ranged from 4 (Chr. 1b) to 38 (Chr. 8). In the genetic map, most of the markers’ orders were collinear to the physical map.

### 2.6. Identification of QTLs Conferring Resistance to GSB in Watermelon

**Figure 3:**
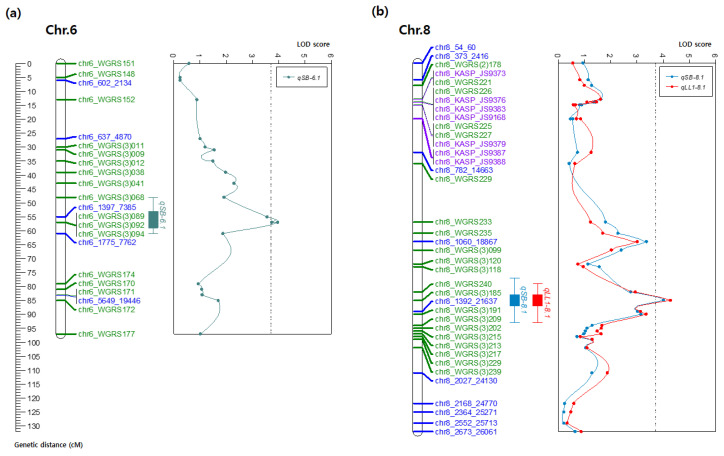
Quantitative trait loci (QTLs) associated with gummy stem blight (GSB) resistance on chromosome 6 and chromosome 8. (**a**) QTLs position of GSB resistance on chromosome 6 of the watermelon genetic map. QTL plot obtained by composite interval mapping (CIM) analysis on chromosome 6. LOD score at peak is 3.96. (**b**) QTLs position of GSB resistance on chromosome 8 of the watermelon genetic map. Major QTLs for stem blight (SB) and leaf lesion (LL) are represented by the blue and red, respectively. QTL plot obtained by composite interval mapping (CIM) analysis on chromosome 8. LOD score at peak for SB and LL is 4.02 and 4.28, respectively.

**Figure 4:**
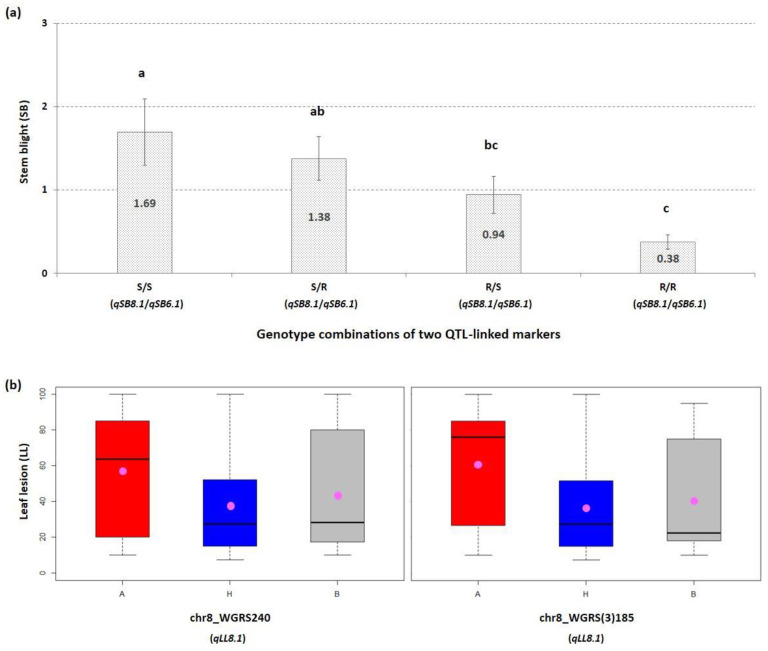
Comparison of GSB resistance in F_2_ population according to genotypes for QTLs *qSB8.1* and *qSB6.1* (**a**) The mean of stem blight (SB) according to genotypes of two QTL-linked markers (chr8_WGRS(3)185 and chr6_WGRS(3)092) where R, resistant genotype and S, susceptible genotype. Bars on graphs indicate standard error and different small letters (a, ab, bc and c) refer to significant differences (*p* < 0.05) according to Duncan multiple range test. (**b**) Box plots of tightly linked SNP markers to QTL for leaf lesion (LL) trait in the F_2_ population. A, genotype of female parent (‘920533’); B, genotype of male parent (‘PI 189225’); H, genotype of heterozygote. The pink dots within the boxplots represent the mean of lines harboring respective alleles.

A total of three QTLs, *qSB6.1*, *qLL8.1*, and *qSB8.1* were identified associated with GSB resistance in 178 individuals of the F_2_ population (Figure 3a,b). Two QTLs located on chromosome 8 were detected as major QTLs and one QTL located on chromosome 6 as a minor QTL (Figure 3a,b and Table 2). The QTLs, *qLL8.1* and *qSB8.1* explained 10.5 and 10.0% of phenotypic variations (*R^2^*, %) for GSB resistance, with LOD scores of 4.28 and 4.02, respectively (Table 2). Interestingly, *qLL8.1* and *qSB8.1* both localized in the same area (85 cM) and sharing the same top markers (20.6 to 21.5 Mbp) on chromosome 8 (Figure 3a,b and Table 2). Whereas, *qSB6.1* on chromosome 6 was detected only for SB trait at 57.1 cM and explained 9.7% of phenotypic variations in GSB resistance with a LOD score of 3.96 (Table 2). Alleles conferring resistance to GSB were contributed by the resistant parent (‘PI 189225’) because additive effect of each QTL were positive (>0) (Table 2). Disease score associated with specific QTL (*qSB8.1* and *qSB6.1*) genotypes were evaluated and compared to determine the independent and combined QTLs genetic effects. A clear trend related to resistance level for GSB was observed with the presence/absence of susceptible (S) and resistance (R) alleles of the QTLs (Figure 4a). The genotype carrying S alleles for both QTL showed reduced resistant level significantly for the SB trait with average score of 1.69 (Figure 4a). The genotypes carrying S allele for *qSB8.1* and R allele *qSB6.1* showed average disease score 1.38 whereas the genotypes carrying R allele for *qSB8.1* and S allele *qSB6.1* showed average disease score 0.94 (Figure 4a). 

When the genotypes carrying R alleles for the both QTLs were compared the average disease score was significantly reduced as up to 0.38 (Figure 4a). These results revealed the additive effect of the two QTL when combine (Figure 4a). For LL trait, boxplot results revealed that the homozygous resistant genotype ‘B’ of *qLL8.1* is associated with increased resistance compared to the homozygous susceptible genotype ‘A’ of *qLL8.1* in the F_2_ population (Figure 4b).

### 2.7. Validation of Flanking Markers Using Watermelon Accessions and Cultivars

A total four markers were developed and validated for GSB-resistant QTL selection. Among them, two markers were developed on chromosome 8 for QTLs (*qLL8.1* and *qSB8.1*) and two on chromosome 6 for QTL (*qSB6.1*) (Table 3). For all these markers, disease severity for SB trait was significantly lower in the individuals having homozygous-resistant alleles R compared to the individuals carrying homozygous susceptible alleles S (Figure 4a). In order to validate QTLs effect of *qLL8.1* and *qSB8.1*, two flanking markers (chr8_WGRS240 and chr8_WGRS(3)185) were converted into KASP™ markers and validated in 9 watermelon accessions and 13 commercial cultivars (Table 3). These two flanking markers tightly linked to *qSB8.1* and *qLL8.1* were found to be associated with resistance to GSB because 6 accessions (*C. amarus*) had high resistance to GSB and possessed resistant genotypes for (KASP_WGRS240 and KASP_WGRS(3)185) (Table 4). To test the utility of the developed KASP™ markers linked to *qSB6.1*, the markers (KASP_WGRS(3)089, KASP_WGRS(3)092) were also validated in 9 accessions and 13 commercial cultivars (Table 4). However, two flanking markers linked to *qSB6.1* didn’t show a very strong correlation with resistance to GSB because ‘PI 279461’ and some commercial cultivars (‘Fair Fax’, ‘Crimson sweet’, and ‘Charleston Gray’) susceptible to GSB held resistant genotypes for (KASP_WGRS(3)089 and KASP_WGRS(3)092). However, these markers in combination can maximize the selection of QTL segments for GSB resistance.

### 2.8. Identification of Candidate Genes for GSB Resistance

Two major QTLs (*qLL8.1* and *qSB8.1*) on chromosome 8, flanked by two markers (chr8_WGRS240 and chr8_WGRS(3)185) (Figure 3b). The interval between these two flanking markers covered approximately 0.87 Mbp on chromosome 8, a region containing 83 genes in Cucurbit Genomics Database (http://cucurbitgenomics.org/; accessed on 15 June 2020) (Table S5). Among the 83 genes, three were encoding receptor-like kinase (RLK) domain-containing proteins and one leucine-rich repeat (LRR) receptor-like protein kinase were considered candidate genes for GSB resistance (Table 5). Three RLK genes (*Cla022133*, *Cla022184*, and *Cla022195*) were located at 825, 219, and 114 kb upstream of the most significant SNP (chr8_WGRS(3)185), respectively (Table 5). An LRR-RLK gene (*Cla022196*) was identified at 111 kb upstream of the highly significant SNP (Table 5). These genes could be potential candidate genes for GSB resistance. However, further studies would confirm their role in GSB resistance in watermelon.

## 3. Discussion

Several different inheritance patterns have been described for controlling GSB resistance in cucurbits, such as a single recessive gene [16,37], single dominant [26,38], and multiple genes [11,17,28,29,37]. Inheritance of resistance to GSB in watermelon was previously described as a single recessive gene, *db*, from ‘PI 189225’ [16]. However, recent research has suggested that several loci might be involved in GSB resistance [15,17]. These discrepancies might be due to the use of different sources of resistance, mapping population, different pathogen isolates and inoculation methods [28]. In the present study, the segregation ratio of F_2_ plants supported that multiple genes are involved in resistance to GSB (Table S1). Two types of disease assessment methods, including leaf lesion (LL) and stem blight (SB), were used for the assessment of accurate phenotypes related to GSB resistance (Figures S5 and S6). Continuous distribution was observed in the present study for the F_2_ population (‘920533’ × ‘PI 189225’) in disease assessment results suggesting the quantitative control of GSB resistance.

*D. bryoniae* can infect the watermelon plants at any stage and on all parts of the plant, including the stem, foliar and fruits. Disease resistance to GSB normally assessed through spraying the inoculum with spore suspension at four to six leaf stage of watermelon seedlings [28,29,35]. Pathogenicity, aggressiveness and environmental factors such as temperature and humidity can influence the resistance evaluation [28]. Variation in the isolates’ aggressiveness, inoculation, post-inoculation disease management practices and disease scoring method can also affect the resistance evaluation and further mapping studies [28,29]. Recently, a research group observed less resistance level of “PI 189225” when kept under 100% relative humidity after inoculation for 48 h compared to 24 h [28]. In the present study, for precise resistance evaluation we used a previously tested aggressive *D. bryoniae* isolate KACC 40937 and evaluated the resistance using two different criteria [8]. These methods could be useful for the separate resistance evaluation on different plant parts as well as comparison of the resistance on foliar and stem in the future studies.

The high-density genetic linkage maps are crucial to detect quantitative trait locus (QTL) for important traits such disease resistance [28,29]. With the advent of high-density molecular genetic linkage maps and advanced mapping technologies, it is achievable to assess the number of QTLs in the genome. Using modern and high-throughput sequencing technology, more genomes sequences are available, thus enabling the development of abundant molecular markers [39]. The earliest SNP markers based maps were developed and compared utilizing three populations in watermelon [40]. The spans of these linkage maps were 1144 cM, 1438 cM, and 1514 cM with average marker intervals of 3.4 cM, 3.8 cM, and 4.2 cM, respectively [40]. SNP markers have improved marker resolution in *Cucurbita pepo*, and melon [41,42]. In our study, we employed two different approaches to obtain SNP markers including NGS-based re-sequencing (Hiseq™ 4000) and Fluidigm^®^ assays. We constructed a genetic linkage map with 188 SNP markers using 104 Fluidigm^®^ assays, 77 HRM markers, and 7 previously reported KASP™ markers, which yielded a genetic linkage map spanning 1070.2 cM. Our linkage map was comparable with the previously developed linkage maps [29,39,40].

QTLs controlling resistance for GSB have been reported in cucurbits such as, cucumber and watermelon [24,25,28,29]. Two QTLs associated to GSB resistance on chromosome 4 (*GSB4*) and 6 (*GSB6b*) at genomic position 12 cM and 11 cM in cucumber (*Cucumis sativus*) were reported using introgression lines [11]. Another research group reported GSB-resistant QTLs using recombinant inbred lines (RILs) and identified total six QTLs on chromosomes 3, 4, 5 and 6 in cucumber [24]. Among those QTLs, a locus *gsb5.1* was repeatedly detected in three seasons and explained 17.9% phenotypic variations [24]. Another QTL analysis reported five QTLs associated to GSB resistance in cucumber using RILs population and major QTL *gsb-s6.2* was mapped on chromosome 6, which explained 22.7% phenotypic variations [25]. A QTL conferring resistance against GSB in watermelon was detected on chromosome 8 (*Qgsb8.1*) using an F_2_ segregating population developed by “PI 189225” as the resistance parents and “K3” as a susceptible parent [28]. *Qgsb8.1* was mapped between two SNP markers KASP_JS9168 and KASP_JS9383 spanning a 0.57 Mb region at physical location of 11,425,655–11,995,922 on chromosome 8 [28]. Another recent study detected three QTLs linked to GSB resistance in watermelon on chromosomes 3, 5 and 7 using F_2:3_ mapping population developed by crossing susceptible line Crimson Sweet and resistant accession PI 482276 [29]. We identified three QTLs on chromosome 6 (*qSB6.1*) and 8 (*qLL8.1* and *qSB8.1*) for LL and SB traits; among them, two QTLs for the SB traits were co-located and shared the common markers. However, the genomic and physical position of the recently detected QTL *Qgsb8.1* was different from the QTLs identified in present study (Table 2) [28]. Although the source of resistance was the same (PI 189225) in both studies but Ren et al. [28] used local *S. cucurbitacearum* (syn. *D. bryoniae*) isolate collected from east China for resistance assessment, whereas we used *D. bryoniae* Korean isolate. Furthermore, the physical position of the QTLs detected by Ren et al. [28] on chromosome 8 corresponded to 11.4 to 11.9 Mbp on the reference genome, whereas we detected QTLs at the genomic position of 20.6 to 21.5 Mbp on chromosome 8. It is yet to be discovered whether the QTLs conferring resistance against different geographic isolates of *Stagonosporopsis* spp. are carrying broad-spectrum resistance. Further fine mapping of the QTLs detected on chromosome 8 is required for a better understanding of the GSB resistance mechanism in watermelon.

Candidate genes for GSB resistance in cucumber and watermelon have been predicted in QTL regions [24,28,29]. One hundred and two potential candidate genes were suggested in the 0.5 cM QTL region in cucumber, and 7 genes associated to disease resistance were reported [24]. In another study of GSB resistance in cucumber, one hundred and seventeen candidate genes were predicted in a QTL region between 3.2 cM genomic distance; among them, 14 were associated to disease resistance [25]. A recently reported QTL, *Qgsb8.1* spans a 571.27 kb region and encompass approximately 19 annotated genes; among them two were related to disease resistance [28]. Another GSB resistance study predicted several candidate genes in three QTL regions on different chromosomes among them *ClCG07G013230*, encoding an Avr9/Cf-9 rapidly elicited disease resistance protein, which comprises a non-synonymous point mutation in the DUF761 domain was predicted as a strong candidate gene for GSB resistance in watermelon [29].

In the present study, two major co-located QTLs (*qLL8.1* and *qSB8.1*) spanned at 0.87 Mbp region on watermelon chromosome 8. A total 83 genes were predicted in QTLs region, out of which four were related to disease resistance. These candidate genes were included three RLK genes (*Cla022133*, *Cla022184*, and *Cla022195*) and one LRR-RLK (*Cla022196*) gene. An overview of reported works related to GSB resistance in cucurbits revealed that mostly candidate resistance genes belongs to NBS-LRR and RLK proteins [37]. We also predicted three RLK genes and one NBS-LRR gene as a candidate gene for GSB resistance in watermelon. However, additional fine mapping, expression analysis and cloning work will be required to pinpoint the candidate gene.

One of the main hurdles in the development of GSB-resistant cultivars is time-consuming difficult phenotyping process and discrepancies in phenotyping results [15,28]. Development and implementation of molecular markers can help in early and precise selection to breed GSB-resistant cultivar through marker-assisted selection in watermelon. In recent studies, high throughput KASP™ assays for GSB resistant has been developed for resistant QTLs selection on chromosome 5, 7 and chromosome 8 [28,29]. We developed and validated four high throughput KASP assays for efficient selection of GSB resistance on chromosome 6 and 8. Two flanking markers (chr8_WGRS240 and chr8_WGRS (3)185) on chromosome 8 linked to QTLs (*qLL8.1* and *qSB8.1*) showed promising results for selection with high marker-trait association, whereas two flanking markers (chr6_WGRS (3)089 and chr6_WGRS (3)092) on chromosome 6 linked to QTL (*qSB6.1*) showed a resistant genotype with watermelon accessions but showed some contrasting genotype with commercial cultivars. However, as the results of QTL–QTL interaction of this study revealed, the presence of both alleles (*qSB6.1* and *qLL8.1*-*qSB8.1*) significantly enhanced the GSB resistance (Figure 4a). These four markers in combination can be utilized to facilitate the marker-assisted selection to incorporate GSB resistance in elite watermelon cultivars.

## 4. Materials and Methods

### 4.1. Plant Materials and DNA Extraction

The GSB-resistant line (*C. amarus* Schrad.; ‘PI 189225’) and GSB-susceptible line (*C. lanatus* (Thunb.) Matsum. & Nakai; ‘920533’) were obtained from Fruit Vegetable Breeding Laboratory of National Institute of Horticultural & Herbal Science (NIHHS, Wanju, South Korea) in 2013. The parental lines were subjected to five rounds of self-pollination to obtain homozygosity. The F_1_ plants were produced by crossing ‘920533’ as the maternal parent with ‘PI 189225’ (paternal parent) in 2014. In May 2017, the F_1_ plants were self-pollinated to produce F_2_ progeny. The F_2_ seeds were sown in November 2017, and 178 F_2_ individuals were used for DNA extraction and disease assessment. Genomic DNA was isolated from susceptible and resistant parents and an F_1_ plant, 178 F_2_ individuals, 9 watermelon accessions, and 13 commercial cultivars using the cetyltrimethylammonium bromide (CTAB) method [43]. Subsequently, 700 μL of 70% ethanol was added, centrifuged for 1 min. at 12,000 rpm, and then the supernatant was removed and the pellet was washed two times. Finally, the DNA was dissolved in 50 μL triple-distilled water (TDW). The concentration of DNA was quantified by NanoVue (GE Healthcare, Chicago, IL, USA) and DNA diluted to 20 ng·L^−1^ was used for further analysis.

### 4.2. Pathogen Inoculation and Disease Assessment

The *D. bryoniae* ‘KACC 40937 isolate’ was collected from Korean Agricultural Culture Collection (KACC) [8]. Potato dextrose agar (PDA) was used to culture *D. bryoniae*. The agar plugs with mycelium were sub-cultured in PDA plates and incubated for five days at 25 °C under alternating periods of 12 h of fluorescent and 12 h of darkness for conidia formation. Spore suspension was prepared by flooding the culture plates with distilled water and filtering the liquid through fine cloth. Spore concentration was measured with a hemocytometer and adjusted to a four × 10^5^ spore · mL^−1^ by adding sterile water. Seedlings with two to three fully expanded leaves were inoculated with *D. bryoniae* by spraying 10 mL spore suspension per plant. The infected plants were incubated in a humidity chamber at 25–27 °C for 48 h and then transferred to a growth chamber at 25 °C with 12 h light a day [4]. 

The disease severities on each parental lines, their F_1_ progenies, 178 individuals of F_2_ population, 9 watermelon accessions, and 13 commercial cultivars were assessed at five days after inoculation (DAI). Briefly, disease assessment was performed using two disease severity indexes. First, leaf lesion (LL) was measured for evaluation of disease severity (Figure S5). Five days after inoculation, LL of all true leaves on each plant was measured in multiples of five. LL percentage (%, 0–100) was scored by calculating the ratio of GSB lesion area to total leaf area. The average lesion of leaves on each plant was calculated. Second, symptom for evaluation of disease severity on stem and petiole were measured (Figure S6). Stem blight (SB) was rated on a scale of 0–3, as follows: 0, no symptoms; 1, moderate symptoms; 2, slight necrosis; 3, severe blight symptoms. Third, assessment using modified ordinal disease rating scale was also conducted [15]. To score the individuals as a resistant and susceptible a disease index (DI, 0–7) was defined by considering the disease severity of LL and SB together, with 0, no symptoms; 1, moderate symptoms (≤20% necrosis) on leaves only; 2, slight symptoms (>45% necrosis) on leaves only; 4, moderate symptoms (≤20% necrosis) on leaves with moderate necrosis also on petiole and stem; 5, slight necrosis also on petiole and stem; 7, severe necrosis also on petiole and stem, death of plant (Table S6). Lastly, plants with a disease index (DI) 0–3 were considered resistant to GSB and plants with a disease index (DI) 4–7 were considered susceptible to GSB (Table S6).

### 4.3. SNP Genotyping Using Fluidigm^®^ SNP Type^TM^ Assays

Fluidigm^®^ genotyping was performed by the Foundation of Agricultural Tech, Commercialization & Transfer (FACT) using the Fluidigm^®^ EP1™ (Fluidigm, San Francisco, CA, USA) high-throughput (HT) genotyping platform. Previously developed 438 Fluidigm^®^ SNP Type™ assays were used to analyze the genotype of F_2_ population [34]. After performing polymorphic survey, genotyping analysis was conducted to 178 individuals of F_2_ population using Fluidigm^®^ SNP Genotyping Analysis version 4.1.3 software.

### 4.4. Next-Generation Re-Sequencing and SNP Detection

The homozygous susceptible ‘920533’ and resistant ‘PI 189225’ parental lines were sequenced through next-generation sequencing by the bioinformatics company Macrogen (Seoul, Korea) [35]. The sequencing libraries were prepared according to the manufacturer’s instructions using TruSeq DNA Nano Sample Preparation Kit (Illumina Inc., San Diego, CA, USA). The whole-genome resequencing was performed using a Hiseq™ 4000 sequencer (Illumina Inc., San Diego, CA, USA). After raw reads from re-sequenced data were aligned to the reference genome, reads were mapped to the reference genome [44] using Burrow-Wheeler Aligner (BWA) version 0.7.17. SNPs using SAMtools version 1.6 software were detected [45,46]. Next, 400-bp flanking sequences (amplicon sequences) of each SNP detected between ‘920533’ and ‘PI 189225’ were obtained. Among these, SNPs were filtered to select those with the following characteristics: (i) homozygous SNPs, (ii) SNPs with A/G, A/C, T/G, and T/C combinations, excluding A/T and G/C, (iii) SNPs with one copy number reference genome as a result of BLASTN version 2.7.1+, and (iv) SNPs on the genic region [35,47].

### 4.5. HRM Primer Design and Genotyping

A total of 888 uniformly distributed HRM primers that amplify sequences for each SNP from NGS data were designed [35,48]. Reactions were conducted in a 20 μL total volume containing 2.0 μL of genomic DNA, 2.0 μL of 10× PCR buffer, 1.0 μL of 2.5 mM dNTP mixture, 0.1 μL of *Taq* DNA polymerase (Takara, Japan), 1.0 μL of SYTO^®^ 9 green-fluorescent nucleic acid stain (Life Technologies™, Carlsbad, CA, USA), 1.0 μL of each primer at a concentration of 10 pmole · mL^−1^ (0.5 μL of each forward and reverse primer) and the rest of total volume was adjusted with TDW. PCR was performed using a PCR machine (Eppendorf, Hamburg, Germany). The PCR cycles were as follows: pre-incubation at 98 °C for 2 min., then 40 cycles: 98 °C for 5 s (denaturation), 60 °C for 10 s (annealing & extension). A HRM analysis was conducted using a CFX96 Touch Real-Time PCR Detection System (Bio-Rad, Hercules, CA, USA) to select polymorphic SNP markers. After PCR, HRM melting curves were displayed by increasing the temperature from 70 °C to 94.6 °C at 0.3 °C intervals. After polymorphic HRM markers based on SNPs were selected using Precision Melt Analysis software (Bio-Rad), two parents, an F_1_ plant, and 178 plants of the F_2_ population were categorized for genotype by three letters (‘A’, ‘H’, ‘B’). Four previously developed HRM markers were also used to analyze genotype in the F_2_ population (Table S3) [36].

### 4.6. Genotyping Using Previously Reported KASP^TM^ Markers

Ten previously reported KASP™ assays (LGC Biosearch™ Technologies, Teddington, UK) were designed for genotyping of the F_2_ population [28,49]. KASP™ assays were performed in a 10 μL reaction volume with 5 μL 2× KASP™ master mix (LGC Biosearch™ Technologies), 0.14 μL assay mix, and 5 μL of 20 ng/μL genomic DNA. The PCR conditions used for the KASP™ assays were as follows: 15 min. at 94°C, followed by 10 cycles of touch down PCR with 20 s 94 °C, 1 min of primer annealing temperature 61 °C with 0.6 °C decrease each cycle, and 26 cycles of 20 s at 94 °C, 1 min at primer annealing temperature, and then 1 min at 37 °C. KASP™ fluorescent end-point readings were measured using a CFX96 Touch Real-Time PCR Detection System (Bio-Rad), and genotype calls were made using Bio-Rad CFX Maestro version 1.1 software (Bio-Rad).

### 4.7. Construction of Linkage Map and QTL Analysis

A genetic linkage map was constructed using JoinMap^®^ version 4.1 software (Kyazma B.V., Wageningen, The Netherlands), with the Kosambi mapping function [50]. Logarithm of odds (LOD) scores from 2.0 to 10.0 and a maximum genetic distance of 5.0 cM were taken as thresholds for the determination of linkage groups (LGs) and genetic distance (GD; cM). Linkage maps were drawn using MapChart version 2.32 software (Wageningen University & Research, Wageningen, The Netherlands) [51]. QTL analysis was performed using the phenotypic data of GSB resistance and genotypic data through marker analysis by MapQTL^®^ version 6.0 software (Kyazma B.V.) [52]. In order to estimate phenotypic variation of each marker, composite interval mapping (CIM) was conducted using the multiple-QTL model (MQM) mapping function. The genome-wide LOD threshold at the 5% significance level was fixed by 1000 permutation tests. QTL analysis was carried out separately using two type of phenotypic data: leaf lesion (LL) and stem blight (SB). Duncan test (*p* < 0.05) was performed to compare between ‘A’, ‘H’, and ‘B’ genotypes of F_2_ individuals using the R *agricolae* package [53].

### 4.8. Validation of Flanking SNP Markers and Identification of Candidate Genes

Nine watermelon accessions (previously reported to have GSB resistance) and 13 commercial cultivars were used for the validation of SNP markers linked to detected QTL regions. The HRM markers within the QTL region were converted into KASP™ markers. KASP™ genotyping assays were designed using Kraken™ Primer Picker software (LGC Biosearch™ Technologies) [49]. The flanking sequences (0.87 Mbp) of QTLs were retrieved from watermelon ‘97103’ genome version 1 in Cucurbit Genomics Database (http://cucurbitgenomics.org/; accessed on 15 June 2020) and candidate genes were predicted in the QTL regions.

## 5. Conclusions

In summary, we performed genetic analyses for gummy stem blight (GSB) resistance using two different phenotyping methods (leaf lesion and stem bight) in F_2_ segregating population of watermelon. We used Fluidigm^®^, HRM, and KASP™ genotyping platforms to analyze SNP markers and constructed a genetic linkage map covering 1070.2 cM with an average marker interval of 5.69 cM. QTL analysis revealed major QTLs, *qLL8.1*, and *qSB8.1*, for LL and SB on chromosome 8, and a minor QTL, *qSB6.1* on chromosome 6, for SB trait only. We suggested four candidate genes associated to GSB resistance that may be key genes for candidate gene analysis in the future. In addition, we developed and validated four high throughput KASP™ assays to facilitate MAS in watermelon for GSB resistance. The finding of the present studies will help to incorporate GSB resistance in elite watermelon cultivars.

## Figures and Tables

**Table 1:** Summary of the distribution of single nucleotide polymorphism (SNP) markers in watermelon genetic map.

Linkage Group	Number of Markers	Map Length (cM)	Marker Density (cM/Marker)	Marker Types			
				Fluidigm^®^ Markers	Previously Reported HRM Markers	Newly Designed HRM Markers	Previously Reported KASP™ Markers
Chr.1a	6	59.1	9.85	1	1	4	-
Chr.1b	4	10.0	2.5	3	-	1	-
Chr.1c	7	39.2	5.6	3	1	3	-
Chr.2a	11	57.8	5.25	10	-	1	-
Chr.2b	7	34.8	4.97	6	-	1	-
Chr.3a	5	21.5	4.3	3	1	1	-
Chr.3b	5	30.2	6.04	4	-	1	-
Chr.4	10	81.4	8.14	4	-	6	-
Chr.5a	5	44.0	8.8	4	-	1	-
Chr.5b	6	44.7	7.45	6	-	-	-
Chr.6	22	97.0	4.41	5	-	17	-
Chr.7	20	98.8	4.94	12	-	8	-
Chr.8	38	131.7	3.47	10	-	21	7
Chr.9	18	134.2	7.46	15	-	3	-
Chr.10	14	89.5	6.39	11	1	2	-
Chr.11	10	96.3	9.63	7	-	3	-
Unmapped	23	-	-	9	-	11	3
Total	211	1070.2	5.69	113	4	84	10

**Table 2:** QTL mapping results for leaf lesion (LL) and stem blight (SB) traits on chromosome 6 and 8 in the F_2_ population.

Trait	QTL	Chr.	Position ^1^ (cM)	Marker Interval	Location (bp)	LOD ^2^	*R^2^* (%) ^3^	Gene Effect	
								Additive Effect	Dominance Effect
Leaf lesion	*qLL8.1*	8	85.376	chr8_WGRS240–chr8_WGRS(3)185	20,663,001–21,535,005	4.28	10.5	9.21	−15.04
Stem blight	*qSB6.1*	6	57.139	chr6_WGRS(3)089–chr6_WGRS(3)092	7,533,583–7,625,669	3.96	9.7	0.07	−0.72
	*qSB8.1*	8	85.263	chr8_WGRS240–chr8_WGRS(3)185	20,663,001–21,535,005	4.02	10.0	0.28	−0.64

^1^ Positions of the markers on the linkage map. ^2^ LOD, logarithm of odds. ^3^ Percent of the phenotypic variation explained by the QTL.

**Table 3:** Primer sequence details of (KASP™) markers associated to GSB resistance.

Marker Name	Chromosome	Location (bp)	Allele	Fluorescent Primer	Sequence (5′-3′)
KASP_WGRS(3)089	6	7,533,583	T	Allele-specific 1 (FAM)	GAATTCAAACTGACATCCAGCACCA
			C	Allele-specific 2 (HEX)	AATTCAAACTGACATCCAGCACCG
			-	Common	GTAACGACGGTCAATCTGTAACGACAA
KASP_WGRS(3)092	6	7,625,669	A	Allele-specific 1 (FAM)	GAGGCAACAGAAGAAGAAGGCAT
			G	Allele-specific 2 (HEX)	GAGGCAACAGAAGAAGAAGGCAC
			-	Common	GAGGCTTATCTTACGTTTCTAGTTCGTTT
KASP_WGRS240	8	20,663,001	A	Allele-specific 1 (FAM)	TGATGAGTAAGAAAAAGAGATTAAAAGCAAAA
			G	Allele-specific 2 (HEX)	GATGAGTAAGAAAAAGAGATTAAAAGCAAAG
			-	Common	GACTCATTTCAAAAGATTTTCTCTGAGGTA
KASP_WGRS(3)185	8	21,535,005	C	Allele-specific 1 (FAM)	ATATGATTCATCTTGGCGGAAACAATG
			A	Allele-specific 2 (HEX)	AAAATATGATTCATCTTGGCGGAAACAATT
			-	Common	TCCAAACCATCATCATCGCTATGACTTA

**Table 4:** Validation of KASP™ markers developed for the gummy stem blight (GSB) resistance in QTL regions.

Watermelon Accessions/Commercial Cultivars			Phenotype	Genotype			
Common Name	Origin	Scientific Name ^1^	Disease Index (DI) ^2^	KASP_WGRS240 (Chr.8)	KASP_WGRS(3)185 (Chr.8)	KASP_WGRS(3)089 (Chr.6)	KASP_WGRS(3)092 (Chr.6)
PI 189225	Congo	*CA*	R	R	R	R	R
‘920533’	South Korea	*CL*	S	S	S	S	S
PI 500335	Zambia	*CA*	R	R	R	R	R
PI 482283	Zimbabwe	*CA*	R	R	R	R	R
PI 164248	Liberia	*CM*	R	S	S	R	-
PI 500334	Zambia	*CA*	R	R	R	R	R
PI 244019	South Africa	*CA*	R	R	R	R	R
PI 482315	Zimbabwe	*CA*	R	R	R	R	R
PI 379243	North Macedonia	*CA*	R	R	R	R	R
PI 279461	Japan	*CL*	S	S	S	R	R
PI 505590	Zambia	*CL*	S	S	S	S	S
‘Fair Fax’	USA	*CL*	S	S	S	R	R
‘Au-Jubilant’	USA	*CL*	S	S	S	S	S
‘Au-Producer’	USA	*CL*	S	S	S	S	S
‘Crimson Sweet’	USA	*CL*	S	S	S	R	R
‘Seupidpeulleoskkul‘	South Korea	*CL*	S	S	S	H	H
‘Heugho‘	South Korea	*CL*	S	S	S	-	S
‘Norangmanidara‘	South Korea	*CL*	S	S	S	S	S
‘Dalgona‘	South Korea	*CL*	S	S	S	H	H
‘Urikkul‘	South Korea	*CL*	S	S	S	S	S
‘Orenjiking‘	South Korea	*CL*	S	S	S	H	H
‘Santakkul‘	South Korea	*CL*	S	S	S	H	H
‘Charleston Gray’	USA	*CL*	S	S	S	R	R
‘Seotaeja‘	South Korea	*CL*	S	S	S	S	S

^1^*CA*, *C. amarus* Schrad.; *CL*, *C. lanatus* (Thunb.) Matsum. & Nakai; *CM*, *C. mucosospermus* (Fursa) Fursa. ^2^ Disease index (DI) was presented as resistant (R, 0–3) and susceptible (S, 4–7).

**Table 5:** Candidate genes associated with gummy stem blight (GSB) resistance and their Gene Ontology (GO) description.

Gene ID	Location		Annotation	SNP (‘920533’/‘PI 189225’)	
	Chr.	Position (bp, Start–End)		Nucleotide	Position (bp)
*Cla022133*	8	20,710,211–20,711,725	Receptor-like protein kinase	A/G	20,710,460
				A/C	20,710,552
				A/G	20,710,776
				C/A	20,710,810
				A/G	20,710,904
				G/A	20,711,132
*Cla022184*	8	21,315,878–21,318,792	Receptor kinase	G/T	21,316,051
				G/A	21,316,141
				T/C	21,316,345
				T/C	21,316,486
				G/A	21,317,089
				C/T	21,317,371
				C/T	21,318,065
				C/T	21,318,362
				T/C	21,318,714
*Cla022195*	8	21,421,183–21,423,645	Receptor kinase	G/T	21,421,391
				G/A	21,421,694
				G/T	21,421,865
				A/G	21,421,905
				T/C	21,422,026
				C/T	21,422,027
				A/G	21,422,704
				T/C	21,422,920
				G/T	21,423,010
				G/A	21,423,031
				C/T	21,423,285
				G/A	21,423,367
				A/G	21,423,644
*Cla022196*	8	21,423,671–21,424,567	Leucine-rich repeat receptor-like protein kinase	T/C	21,423,751
				T/C	21,423,821
				C/T	21,423,899
				C/T	21,423,995
				C/T	21,424,071
				A/C	21,424,242
				A/G	21,424,352
				G/A	21,424,355
				G/T	21,424,503

## Supplementary Materials

The following are available online at https://www.mdpi.com/2223-7747/10/3/500/s1, Figure S1: Gummy stem blight (GSB) reactions on the leaves of resistant parent ‘PI 189225’, susceptible parent ‘920533’, and F_1_, Figure S2: SNP genotyping using Fluidigm^®^ SNP Type™ assays for two parents, F_1_, and 178 individuals of the F_2_ population, Figure S3: Melting curves of 88 polymorphic HRM markers for two parents, F_1_, and 178 individuals of the F_2_ population, Figure S4: SNP genotyping of KASP™ markers for two parents, F_1_, 178 individuals of the F_2_ population, Figure S5: Evaluation of disease severity using leaf lesion (LL) area, Figure S6: Symptoms caused by *Didymella bryoniae* on stem and petiole of test plants, Table S1: Inheritance of resistance to GSB in parental lines, F_1_ progenies and F_2_ population, Table S2: Summary of single nucleotide polymorphisms (SNPs) between watermelon lines ‘920533’ and ‘PI 189225’, Table S3: List of 88 polymorphic SNPs between ‘920533’ and ‘PI 189225’ developed into HRM marker, Table S4: Details of SNPs markers used for the genetic map construction in this study, Table S5: List of genes and their annotation in a QTL regions on chromosome 8, Table S6: Disease index (DI) scale for distinguishing resistance and susceptible plants to GSB.

## Abbreviations

BFB	Bacterial Fruit Blotch
BLASTN	Basic Local Alignment Search Tool Nucleotide
BSA	Bulked Segregant Analysis
CAPS	Cleaved Amplified Polymorphic Sequence
CIM	Composite interval mapping
DAI	Days After Inoculation
dCAPS	derived Cleaved Amplified Polymorphic Sequence
DI	Disease Index
GBS	Genotyping-by-Sequencing
GO	Gene Ontology
GSB	Gummy Stem Blight
HRM	High Resolution Melting
HT	High-throughput
KASP	Kompetitive Allele Specific PCR
LG	Linkage Group
LL	Leaf Lesion
LOD	Logarithm of Odds
LRR	Leucine-Rich Repeat
MAS	Marker-assisted Selection
NGS	Next Generation Sequencing
PRSV-W	Papaya Ringspot Virus-Watermelon (Strain)
QTL	Quantitative trait locus
RAPD	Random Amplified Polymorphic DNA
RIL	Recombinant Inbred Line
RLK	Receptor-Like Kinase
SB	Stem Blight
SCAR	Sequence Characterized Amplified Region
SNP	Single Nucleotide Polymorphism
SSR	Simple Sequence Repeat

## References

[1] Food and Agriculture Organization FAOSTAT. http://www.fao.org/faostat/en/#data/.

[2] Soteriou G., Kyriacou M., Siomos A., Gerasopoulos D. (2014). Evolution of watermelon fruit physicochemical and phytochemical composition during ripening as affected by grafting. Food Chem..

[3] Kim J.Y., Lee S.H., Hwang S.J., Kim G.H., Eun J.-B. (2013). Physicochemical characteristics and functional com-ponents of Mudeungsan watermelon and the other cultivars from Korea. Korean J. Food Sci. Technol..

[4] Lee J.H., Jang K.S., Choi Y.H., Kim J.-C., Choi G.J. (2016). Development of an efficient screening system for re-sistance of watermelon plants to *Didymella bryoniae*. Res. Plant. Dis..

[5] Shim C.K., Seo I.K., Jee H.J., Kim H.K. (2006). Genetic diversity of *Didymella bryoniae* for RAPD profiles substantiated by SCAR marker in Korea. Plant. Pathol. J..

[6] Skarshaug A.J. (1981). Centrum development in *Didymella bryoniae*. Am. J. Bot..

[7] Maynard D.N., Hopkins D.L. (1999). Watermelon Fruit Disorders. HortTechnology.

[8] Kwon M.K., Hong H.J., Sung K.Y., Cho B.H., Kim K.L. (1997). Standardization of a mass-production technique for pycnidiospores of *Didymella bryoniae*, gummy stem blight fungus of Cucurbits. Korea J. Plant. Pathol..

[9] Dos Santos G.R., Sousa S.C.R., Juliatti F.C., Rodrigues A.C., Dalcin M.S., Bonifácio A. (2016). Control of gummy stem blight in watermelon through different management systems. Biosci. J..

[10] Wolukau J.N., Zhou X.-H., Li Y., Zhang Y.-B., Chen J.-F. (2007). Resistance to gummy stem blight in melon (*Cucumis melo* L.) germplasm and inheritance of resistance from plant introductions 157076, 420145, and 323498. HortScience.

[11] Lou L., Wang H., Qian C., Liu J., Bai Y., Chen J. (2013). Genetic mapping of gummy stem blight (*Didymella bryoniae*) resistance genes in Cucumis sativus-hystrix introgression lines. Euphytica.

[12] Norton J.D., Boyan G., Smith D.A., Abrahams B.R. (1995). ‘AU-Sweet Scarlet’ watermelon. HortScience.

[13] Sowell G. (1975). An additional source of resistance to gummy stem blight in watermelon. Plant. Dis. Rep..

[14] Sowell G., Pointer G.R. (1962). Gummy stem blight resistance introduced watermelons. Plant. Dis. Rep..

[15] Gusmini G., Song R., Wehner T.C. (2005). New sources of resistance to gummy stem blight in watermelon. Crop. Sci..

[16] Norton J.D. (1979). Inheritance of resistance to gummy stem blight caused by *Didymella bryoniae* in watermelon. HortScience.

[17] Gusmini G., Rivera-Burgos L.A., Wehner T.C. (2017). Inheritance of resistance to gummy stem blight in watermelon. HortScience.

[18] Branham S.E., Levi A., Katawczik M.L., Wechter W.P. (2019). QTL mapping of resistance to bacterial fruit blotch in *Citrullus amarus*. Theor. Appl. Genet..

[19] Jang Y.J., Seo M., Hersh C.P., Rhee S.-J., Kim Y., Lee G.P. (2018). An evolutionarily conserved non-synonymous SNP in a leucine-rich repeat domain determines anthracnose resistance in watermelon. Theor. Appl. Genet..

[20] Fall L.A., Clevenger J., McGregor C. (2018). Assay development and marker validation for marker assisted selection of *Fusarium oxysporum* f. sp. *niveum* race 1 in watermelon. Mol. Breed..

[21] Branham S.E., Wechter W.P., Lambel S., Massey L., Ma M., Fauve J., Farnham M.W., Levi A. (2018). QTL-seq and marker development for resistance to *Fusarium oxysporum* f. sp. *niveum* race 1 in cultivated watermelon. Mol. Breed..

[22] Branham S.E., Wechter W.P., Ling K.-S., Chanda B., Massey L., Zhao G., Guner N., Bello M., Kabelka E., Fei Z. (2020). QTL mapping of resistance to *Fusarium oxysporum* f. sp. *niveum* race 2 and *Papaya ringspot* virus in *Citrullus amarus*. Theor. Appl. Genet..

[23] Branham S.E., Levi A., Farnham M.W., Wechter W.P. (2017). A GBS-SNP-based linkage map and quantitative trait loci (QTL) associated with resistance to Fusarium oxysporum f. sp. niveum race 2 identified in *Citrullus lanatus* var. citroides. Theor. Appl. Genet..

[24] Liu S., Shi Y., Miao H., Wang M., Li B., Gu X., Zhang S. (2017). Genetic analysis and QTL mapping of resistance to gummy stem blight in *Cucumis sativus* seedling stage. Plant. Dis..

[25] Zhang S., Liu S., Miao H., Shi Y., Wang M., Wang Y., Li B., Gu X. (2017). Inheritance and QTL mapping of resistance to gummy stem blight in cucumber stem. Mol. Breed..

[26] Hu Z., Deng G., Mou H., Xu Y., Chen L., Yang J., Zhang M. (2018). A re-sequencing-based ultra-dense genetic map reveals a gummy stem blight resistance-associated gene in *Cucumis melo*. DNA Res..

[27] Hassan Z., Rahim A., Natarajan S., Robin A.H.K., Kim H.-T., Park J.-I., Nou I.-S. (2018). Gummy stem blight resistance in melon: Inheritance pattern and development of molecular markers. Int. J. Mol. Sci..

[28] Ren R., Xu J., Zhang M., Liu G., Yao X., Zhu L., Hou Q. (2020). Identification and molecular mapping of a gummy stem blight resistance gene in wild watermelon (*Citrullus amarus*) Germplasm PI 189225. Plant. Dis..

[29] Gimode W., Bao K., Fei Z., McGregor C. (2021). QTL associated with gummy stem blight resistance in watermelon. Theor. Appl. Genet..

[30] Kim K.-H., Hwang J.-H., Han D.-Y., Park M., Kim S., Choi D., Kim Y., Lee G.P., Kim S.-T., Park Y.-H. (2015). Major quantitative trait loci and putative candidate genes for powdery mildew resistance and fruit-related traits revealed by an intraspecific genetic map for watermelon (*Citrullus lanatus* var. lanatus). PLoS ONE.

[31] Han B.K., Rhee S.J., Jang Y.J., Sim T.Y., Kim Y.J., Park T.S., Lee G.P. (2016). Identification of a causal pathogen of watermelon powdery mildew in Korea and development of a genetic linkage marker for resistance in water-melon (*Citrullus lanatus*). Korean J. Hortic. Sci..

[32] Ling K.-S., Harris K.R., Meyer J.D.F., Levi A., Guner N., Wehner T.C., Bendahmane A., Havey M.J. (2009). Non-synonymous single nucleotide polymorphisms in the watermelon eIF4E gene are closely associated with resistance to Zucchini yellow mosaic virus. Theor. Appl. Genet..

[33] Wittwer C.T., Reed G.H., Gundry C.N., Vandersteen J.G., Pryor R.J. (2003). High-resolution genotyping by amplicon melting analysis using LCGreen. Clin. Chem..

[34] Park S.-W., Kim K.-T., Kang S.-C., Yang H.-B. (2016). Rapid and practical molecular marker development for rind traits in watermelon. Hortic. Environ. Biotechnol..

[35] Lee E.S., Kim J., Hong J.P., Kim D.-S., Kim M., Huh Y.-C., Back C.-G., Lee J., Lee H.-E. (2018). Development of HRM markers based on SNPs identified from next generation resequencing of susceptible and resistant parents to gummy stem blight in watermelon. Korean J. Breed. Sci..

[36] Lee H.-E., Hong J.P., Suh H.Y., Huh Y.-C., Ahn Y.-K., Kim J., Kim D.-S. (2015). Survey of SNP markers based on genome related to gummy stem blight resistance in watermelon. J. Agric. Sci. Chungbuk Natl. Univ..

[37] Hassan Z., Rahim A., Jung H.-J., Park J.-I., Kim H.-T., Nou I.-S. (2019). Genome-wide characterization of NBS-encoding genes in watermelon and their potential association with gummy stem blight resistance. Int. J. Mol. Sci..

[38] Zuniga T.L., Jantz J.P., Zitter T.A., Jahn M.K. (1999). Monogenic dominant resistance to gummy stem blight in two melon (*Cucumis melo*) accessions. Plant. Dis..

[39] Cheng Y., Luan F., Wang X., Gao P., Zhu Z., Liu S., Baloch A.M., Zhang Y. (2016). Construction of a genetic linkage map of watermelon (*Citrullus lanatus*) using CAPS and SSR markers and QTL analysis for fruit quality traits. Sci. Hortic..

[40] Sandlin K., Prothro J., Heesacker A., Khalilian N., Okashah R., Xiang W., Bachlava E., Caldwell D.G., Taylor C.A., Seymour D.K. (2012). Comparative mapping in watermelon [Citrullus lanatus (Thunb.) Matsum. et Nakai]. Theor. Appl. Genet..

[41] Esteras C., Gomez P., Monforte A.J., Blanca J., Vicente-Dolera N., Roig C., Nuez F., Pico B. (2012). High-throughput SNP genotyping in Cucurbita pepo for map construction and quantitative trait loci mapping. BMC Genom..

[42] Deleu W., Esteras C., Roig C., González-To M., Fernandez-Silva I., Gonzalez-Ibeas D., Blanca J., Aranda M.A., Arús P., Nuez F. (2009). A set of EST-SNPs for map saturation and cultivar identification in melon. BMC Plant. Biol..

[43] Doyle J.J., Doyle J.L. (1990). Isolation of plant DNA from fresh tissue. Focus.

[44] Guo S., Zhang J., Sun H., Salse J., Lucas W.J., Zhang H., Zheng Y., Mao L., Ren Y., Wang Z. (2013). The draft genome of watermelon (*Citrullus lanatus*) and resequencing of 20 diverse accessions. Nat. Genet..

[45] Li H. (2011). A statistical framework for SNP calling, mutation discovery, association mapping and population genetical parameter estimation from sequencing data. Bioinformatics.

[46] Li H., Durbin R. (2009). Fast and accurate short read alignment with Burrows-Wheeler transform. Bioinformatics.

[47] Zhang Z., Schwartz S., Wagner L., Miller W. (2000). A greedy algorithm for aligning DNA sequences. J. Comput. Biol..

[48] Untergasser A., Cutcutache I., Koressaar T., Ye J., Faircloth B.C., Remm M., Rozen S.G. (2012). Primer3—new capabilities and interfaces. Nucleic Acids Res..

[49] He C., Holme J., Anthony J., Fleury D., Whitford R. (2014). SNP genotyping: The KASP Assay. Crop Breeding.

[50] Kosambi D.D. (1943). The estimation of map distances from recombination values. Ann. Eugen..

[51] Voorrips R.E. (2002). MapChart: Software for the graphical presentation of linkage maps and QTLs. J. Hered..

[52] Ooijen J., Kyazma B. (2009). MapQTL 6.

[53] de Mendiburu F. Package ‘Agricolae’. https://CRAN.R-project.org/package=agricolae.

