# Supplementary material for: QTL Mapping for Gummy Stem Blight Resistance in Watermelon (Citrullus spp.)
Source: Plants (Basel). 2021 Mar 8;10(3):500. doi: 10.3390/plants10030500 (PMC7999335; doi:10.3390/plants10030500)
Supplement: Supplementary file 1 [file plants-10-00500-s001.zip › suppli/Supplementary Figure.pdf]

QTL Mapping for Gummy Stem Blight Resistance in Watermelon (*Citrullus* spp.)

Eun Su Lee [1], Do-Sun Kim [1], Sang Gyu Kim [1], Yun-Chan Huh [2], Chang-Gi Back [3], Ye-Rin Lee [1], Muhammad Irfan Siddique [1], Koeun Han [1], Hye-Eun Lee [1,*] and Jundae Lee [4,*]

*****Correspondence: helee72@korea.kr; Tel.: +82-063-238-6674; Fax: +82-063-238-6605 (H.-E.L.); ajfall@jbnu.ac.kr; Tel.: +82-063-270-2560 (J.L.)

Supplementary Material

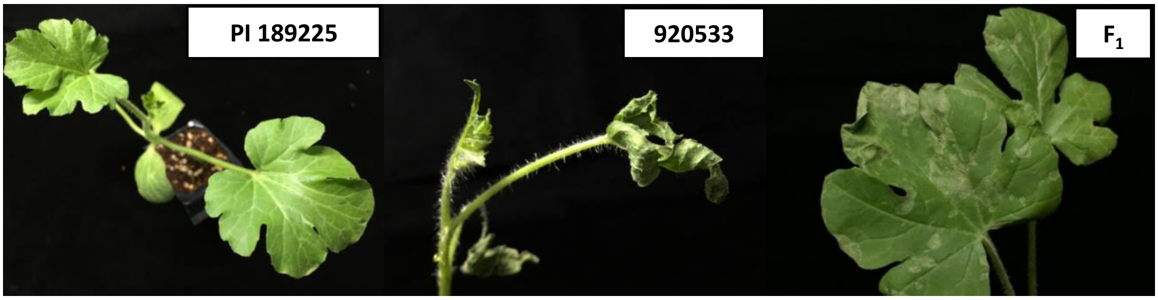

Figure S1. Gummy stem blight (GSB) reactions on the leaves of resistant parent 'PI 189225', susceptible parent '920533', and F_1.

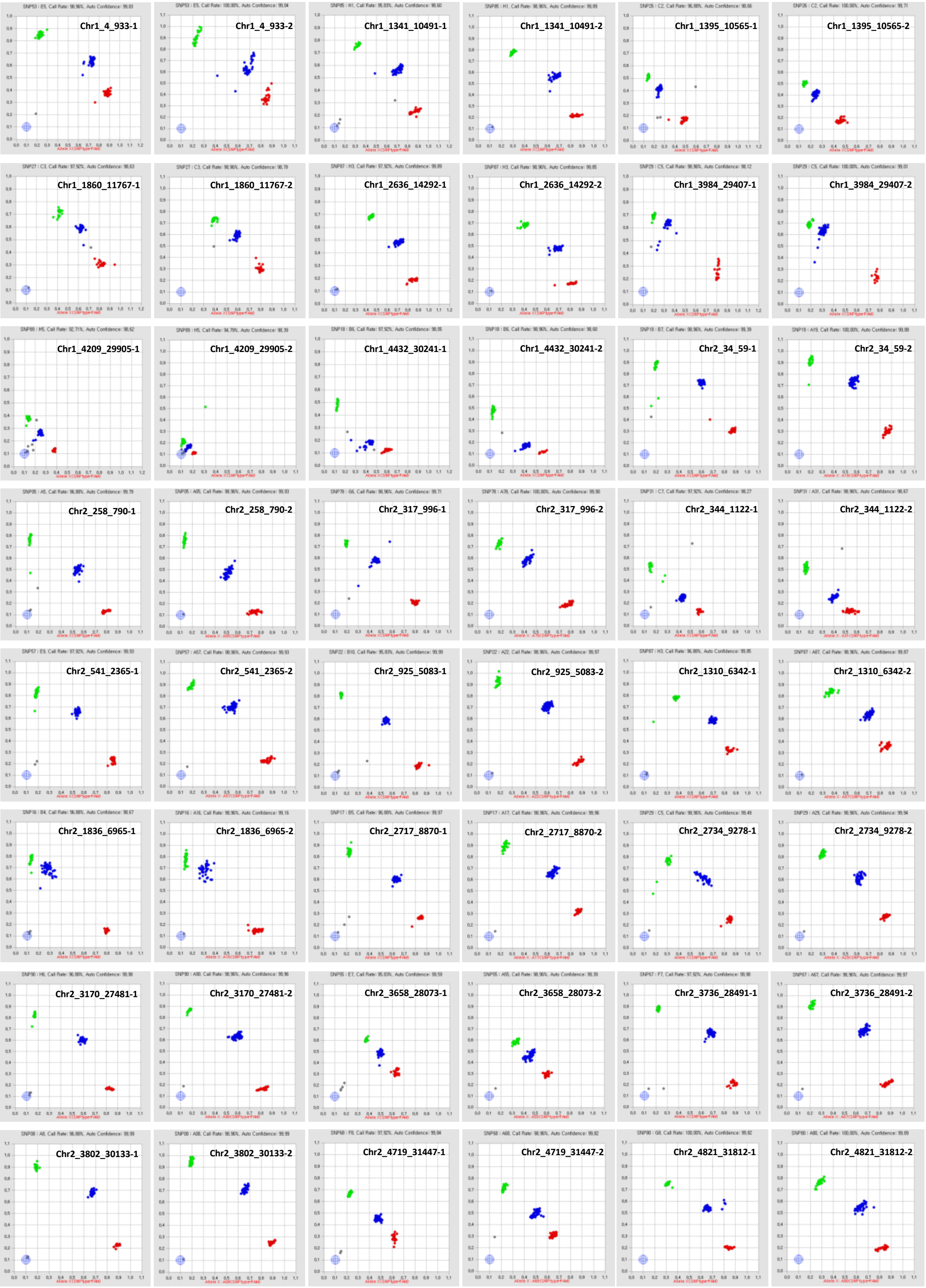

Figure S2. SNP genotyping using Fluidigm® SNP Type™ assays for two parents, F_1, and 178 individuals of the F_2 population. chr1_4_933-1 and chr1_4_933-2 represent scatter plotting of a chr1_4_933 assays for F_2 individuals from 1 to 89 and from 90 to 178, respectively.

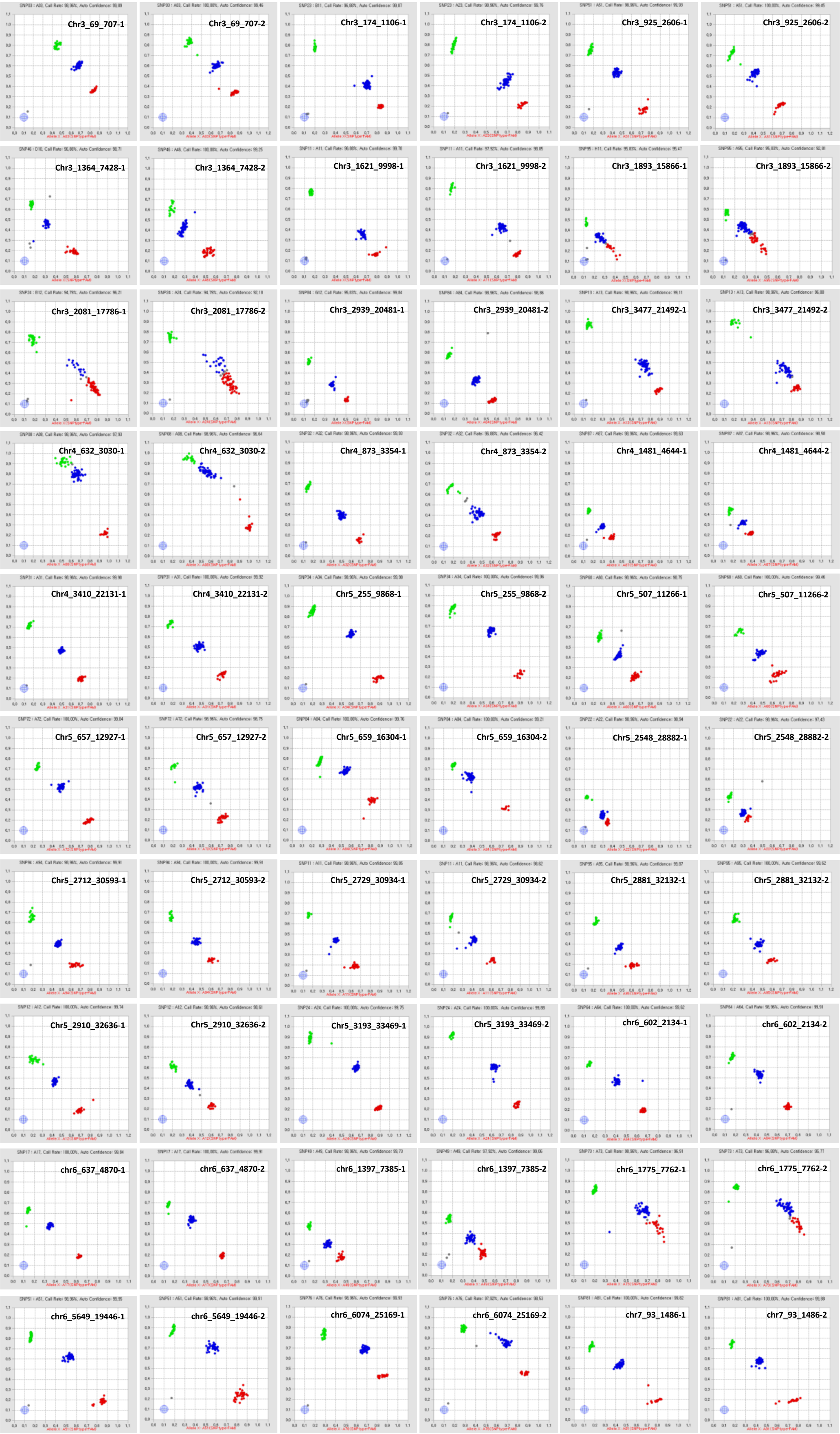

Figure S2. *Cont.*

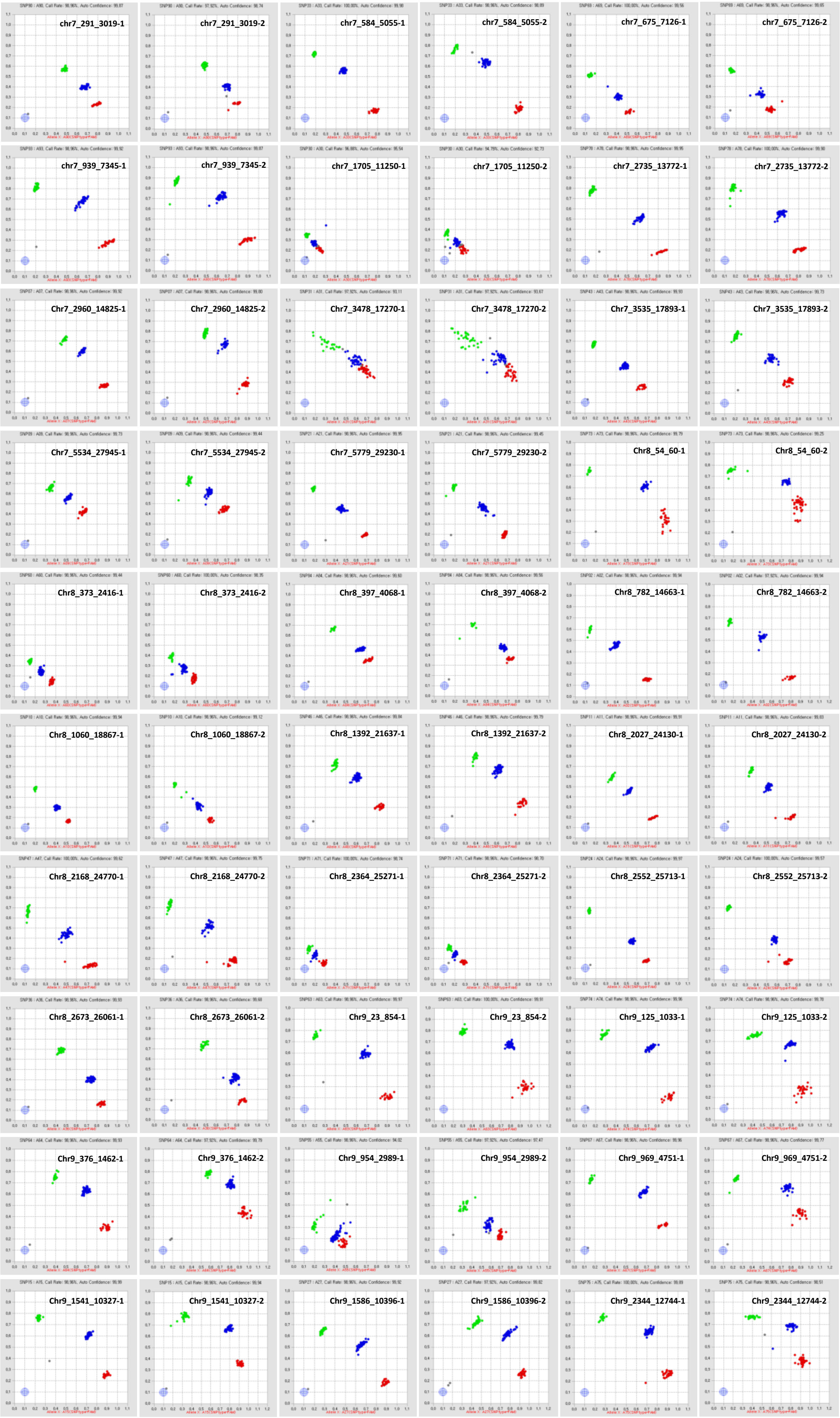

Figure S2. *Cont.*

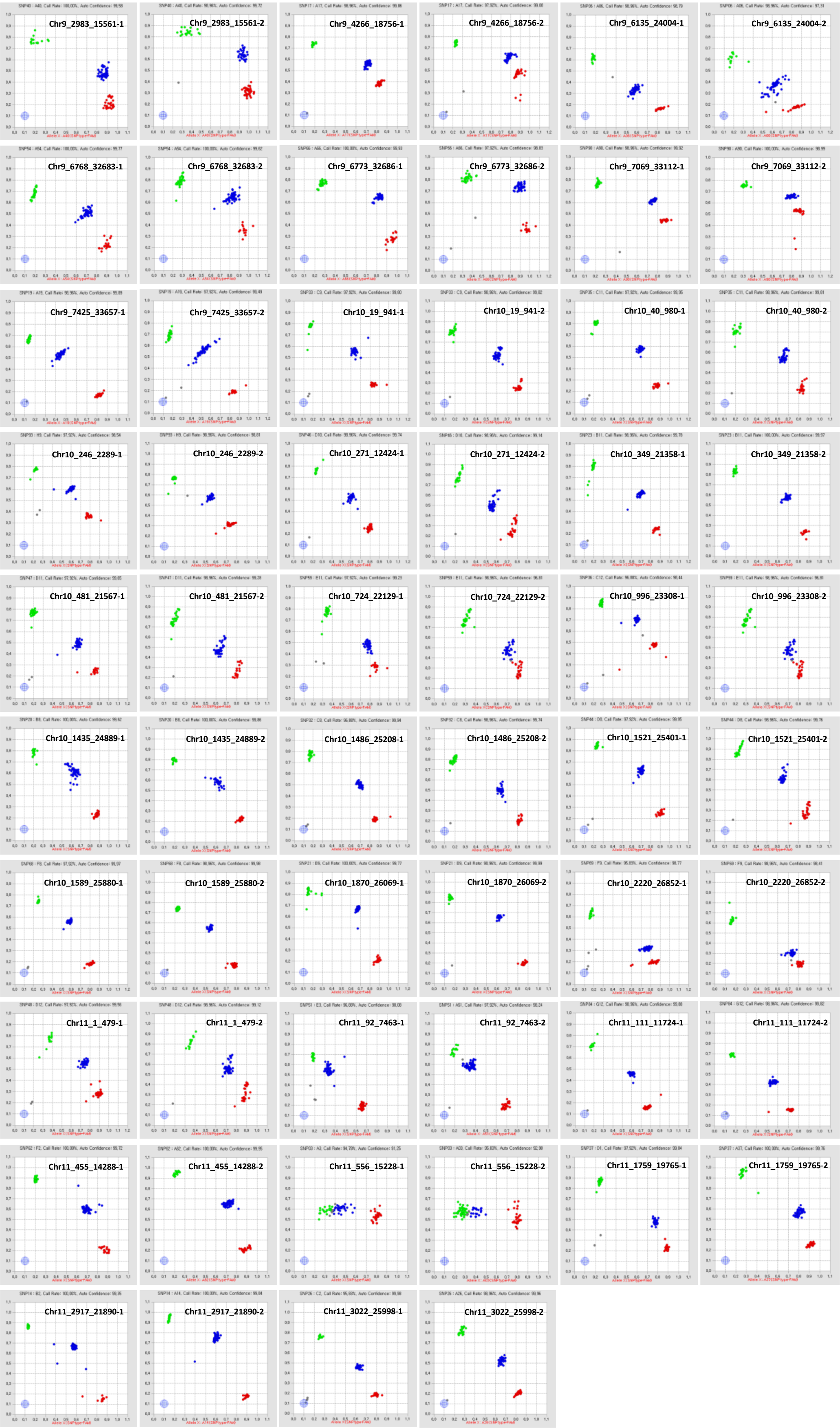

Figure S2. *Cont.*

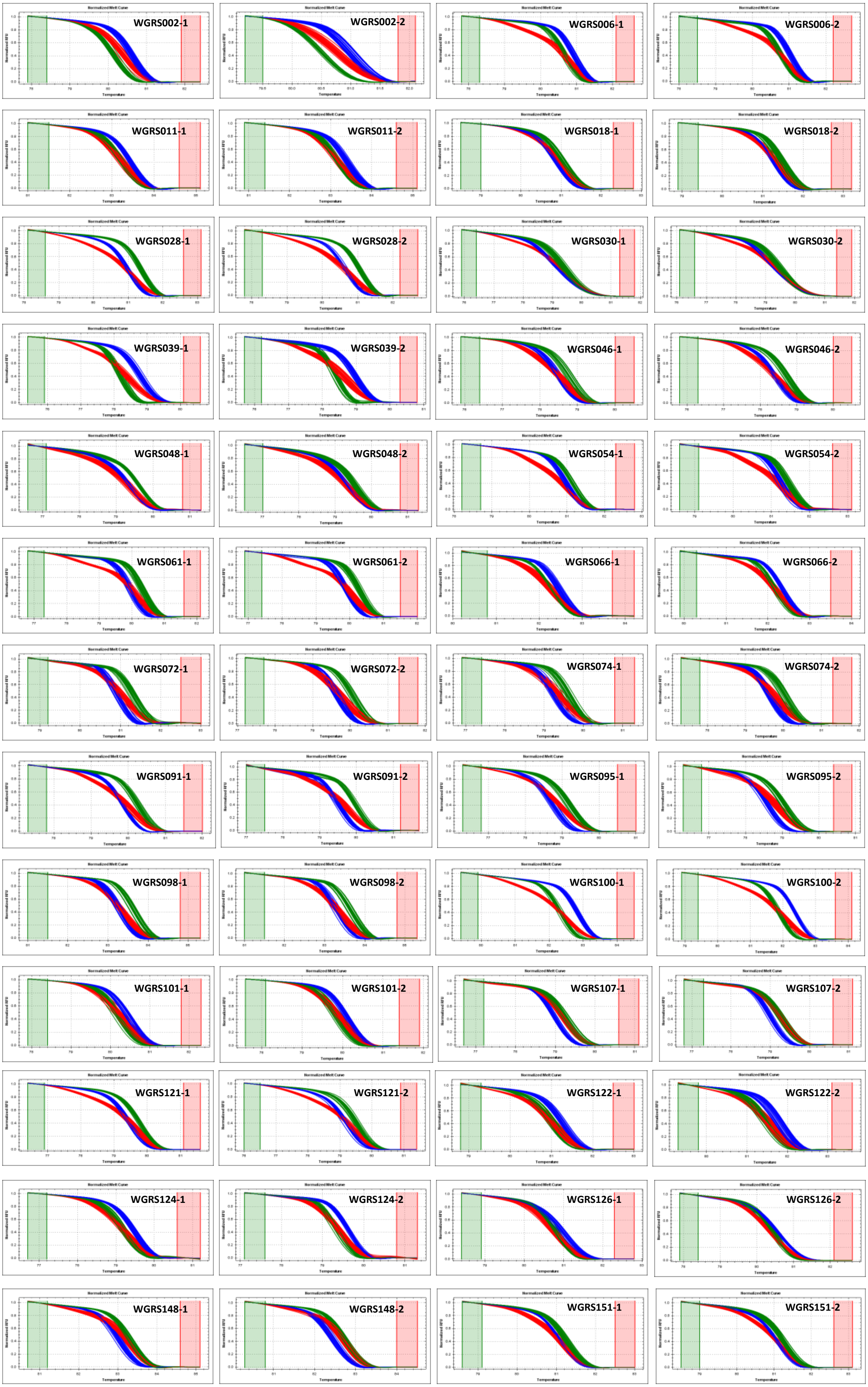

Figure S3. Melting curves of 88 polymorphic HRM markers for two parents, F_1, and 178 individuals of the F_2 population. WGRS002-1 and WGRS002-2 represent melting curves of a WGRS002 HRM marker for F_2 individuals from 1 to 89 and from 90 to 178, respectively.

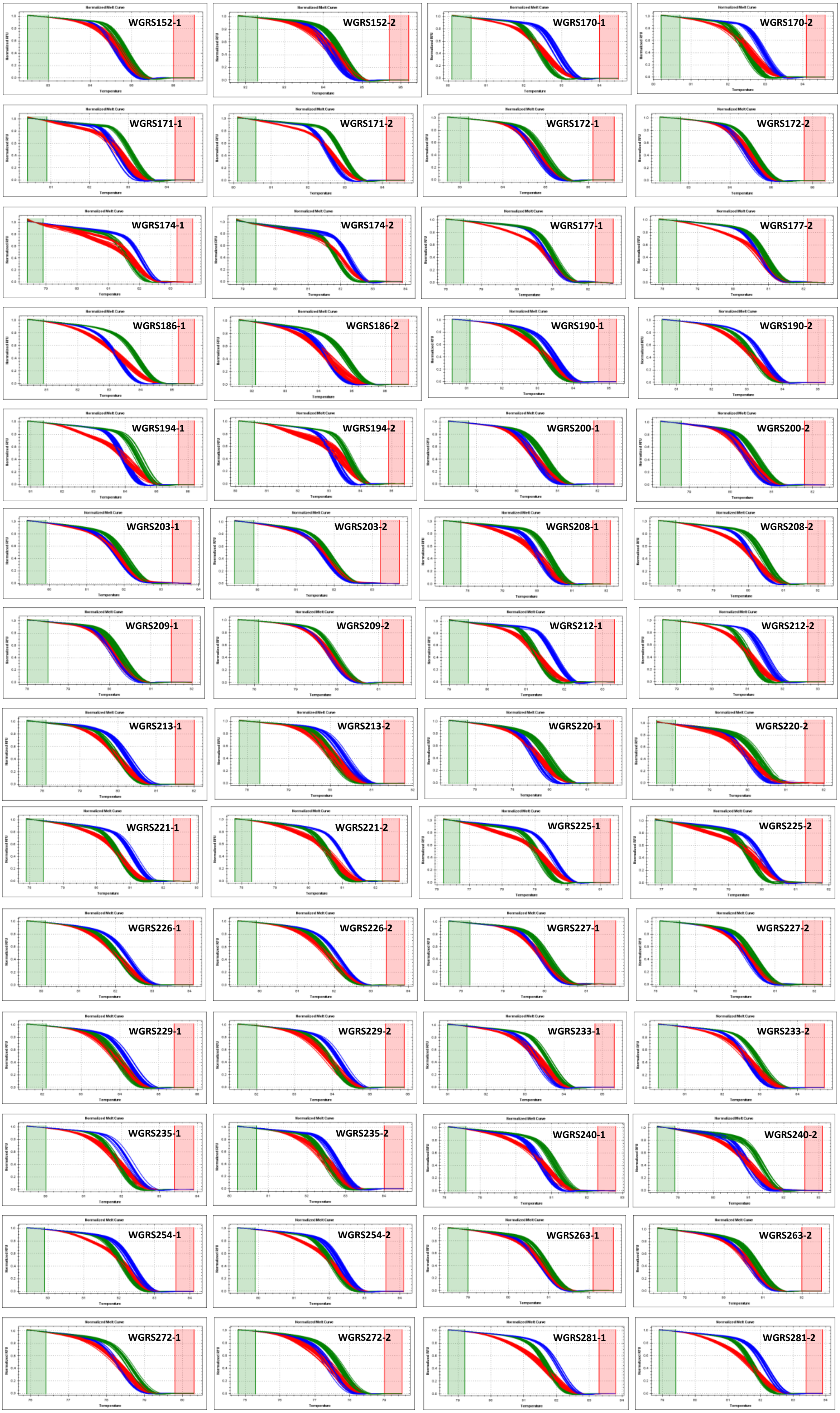

Figure S3. *Cont.*

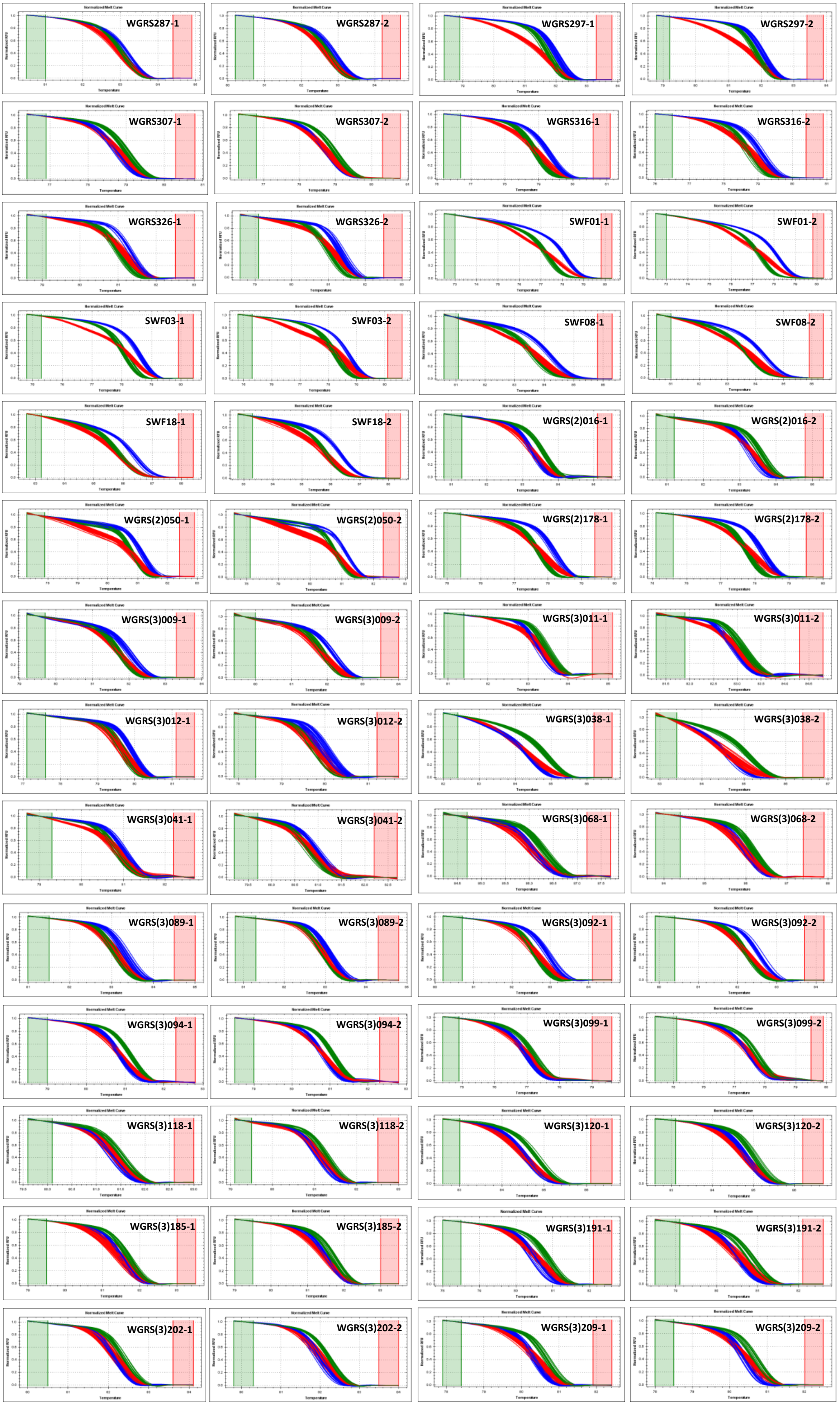

Figure S3. *Cont.*

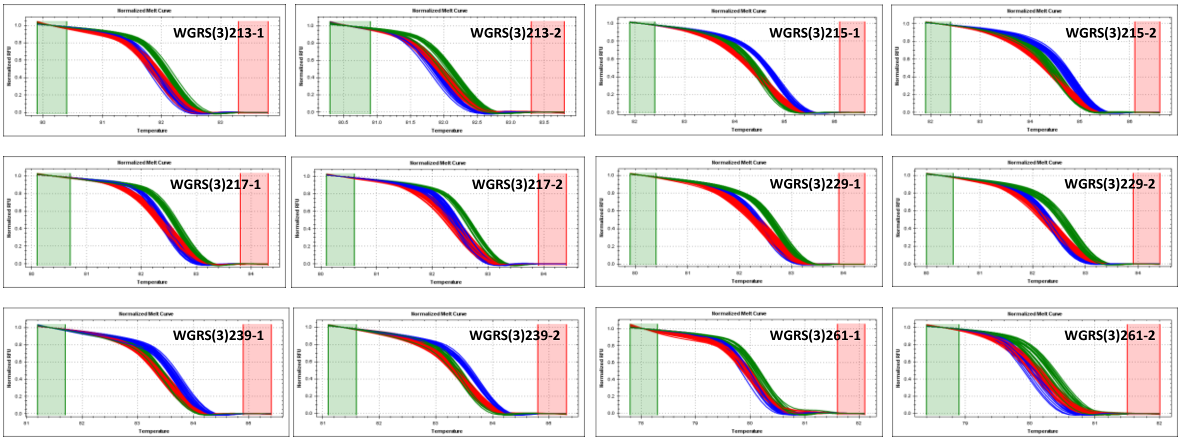

Figure S3. *Cont.*

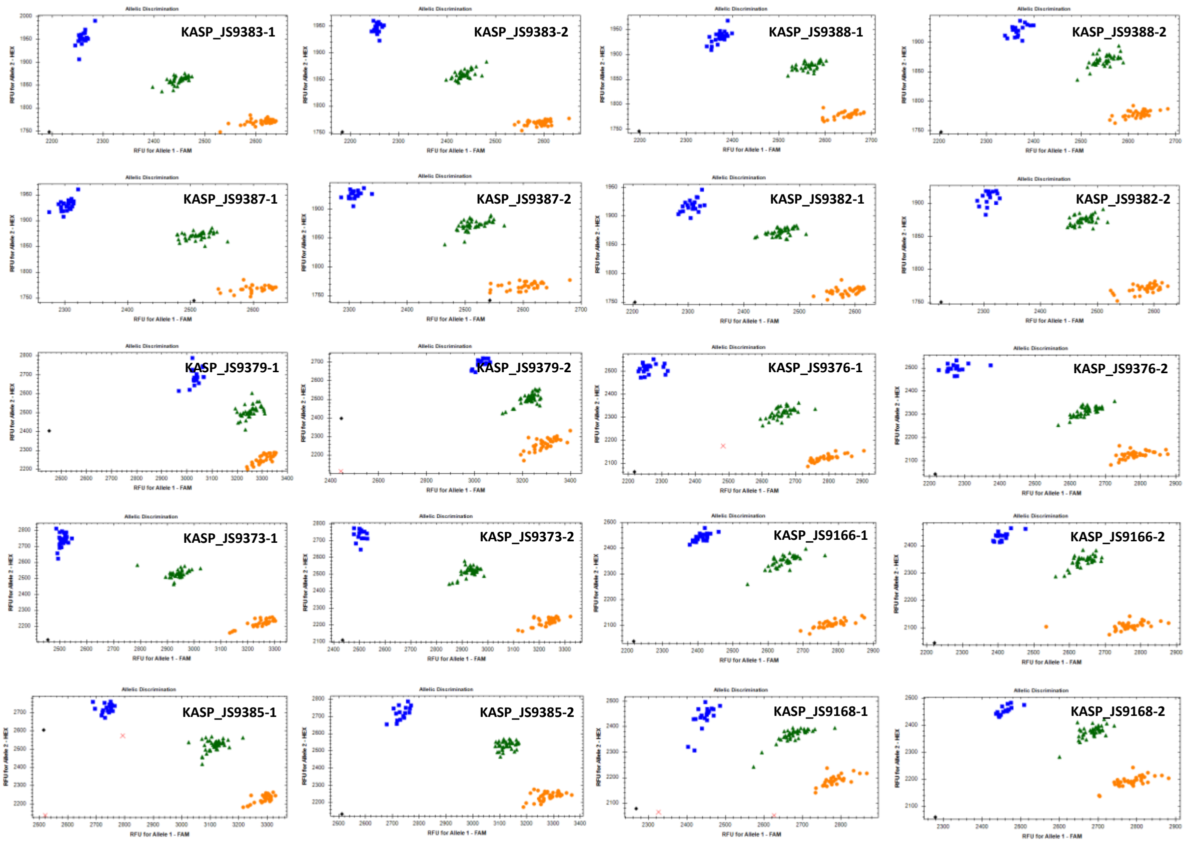

Figure S4. SNP genotyping of KASP™ markers for two parents, F_1, 178 individuals of the F_2 population. Genotyping of previously reported 10 KASP makers to construct a genetic linkage map. KASP_JS9383-1 and KASP_JS9383-2 represent scatter plotting of a KASP_JS9383 marker for F_2 individuals from 1 to 89 and from 90 to 178, respectively.

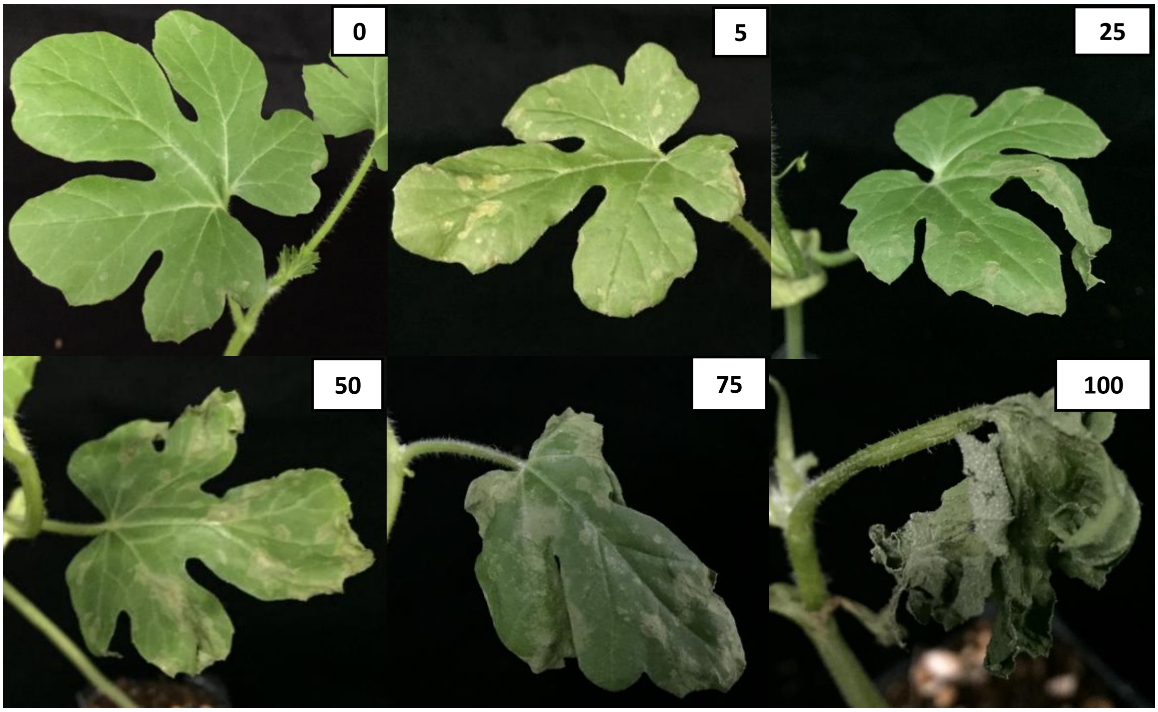

Figure S5. Evaluation of disease severity using leaf lesion (LL) area. The leaf lesion (%) was measured by calculating the ratio of GSB lesion area to the total leaf area on infected plants. Where 0, no leaf lesion; 5, 5% leaf lesion; 25, 25% leaf lesion; 50, 50% leaf lesion; 75, 75% leaf lesion; 100, 100% leaf lesion.

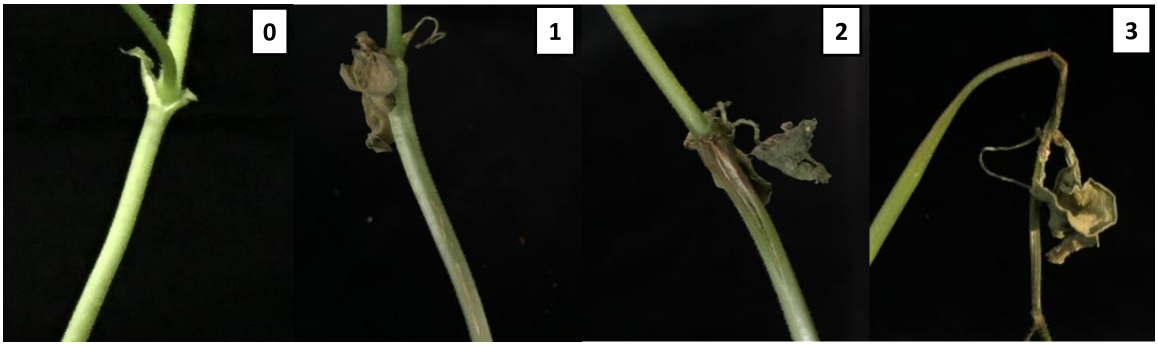

Figure S6. Symptoms caused by *Didymella bryoniae* on stem and petiole of test plants. Stem blight (SB) was scored at a scale of 0~3: Where 0, no symptoms; 1, moderate symptoms; 2, slight necrosis; 3, severe blight symptoms.
